# Opioid deprescribing: rethinking policies to facilitate better patient outcomes

**DOI:** 10.1080/17581869.2025.2516409

**Published:** 2025-06-09

**Authors:** Aili V. Langford, Kellia Chiu

**Affiliations:** a Sydney Pharmacy School, Faculty of Medicine and Health, The University of Sydney, Sydney, Australia; b Centre for Medicine Use and Safety, Faculty of Pharmacy and Pharmaceutical Sciences, Monash University, Parkville, Australia; c Department of Family and Community Medicine, Temerty Faculty of Medicine, University of Toronto, Toronto, Canada

**Keywords:** Opioids, deprescribing, health policy, pain, analgesia

## Abstract

Deprescribing, the patient-centered process of reducing or stopping a medication when the potential harms outweigh the likely benefits, has emerged as a promising strategy to mitigate opioid-related harm. Typically, opioid deprescribing occurs at the individual level, however, adopting a policy-driven approach could expand its reach and impact. To date, prescription opioid control policies that have been implemented with the intention of reducing opioid use and harm have often resulted in unintended consequences. In this article we discuss whether and how the concept of opioid deprescribing can be operationalized at a policy level. We review the goals, challenges and consequences of opioid control policies, explore how they intersect with system-level factors, and propose pathways for developing and implementing future opioid deprescribing policies. We argue that the development and implementation of patient-centered opioid deprescribing policies are both essential and feasible, if key challenges such as structural stigma and the complex interplay between pain and opioid use disorder are recognized and addressed. Robust evaluation frameworks will also be critical for monitoring outcomes and refining interventions. By prioritizing patient and provider needs, and carefully considering pertinent system-level factors, policymakers may be able to foster more effective and compassionate opioid management and reduce opioid-related harm.

## Plain Language Summary

Deprescribing means safely reducing or stopping a medication when its risks are greater than its benefits. Patients and healthcare providers usually make deprescribing decisions together, but the process can be difficult, especially for medications like opioids. This article explores whether opioid deprescribing can be supported not just by patients and healthcare professionals, but also through policies. Some policies have tried to reduce opioid use, but they have sometimes caused unexpected problems. We review what these policies aim to achieve, the challenges they bring, and how they connect to the wider healthcare system. We also suggest ways that future policies can better support opioid deprescribing with a focus on patients’ needs. Effective policies must address key issues like stigma around opioid use, and the link between pain and addiction. Finally, we highlight the importance of assessing whether new policies work as they are meant to. With thoughtful planning, policymakers may be able to create more compassionate and effective strategies to reduce harms from opioids.

## 1. The opioid deprescribing landscape

Opioids are a class of medication used for a range of indications, including pain, dyspnea, restless leg syndrome and opioid use disorder (OUD). Their propensity for harm, whether taken as prescribed or non-medically, has resulted in their classification as high-risk medications [1], with many opioids subject to international control [2]. In recent decades, the prescription and use of opioids have risen markedly in the Global North [3,4], accompanied by increased opioid-related morbidity and mortality [5–7]. In response to what has been described as a *public health crisis* [8,9], many jurisdictions have implemented policies targeting drivers of opioid overuse, including legislation to remove selected opioids and formulations from public drug formularies, instituting prescription drug monitoring programs, and the development and implementation of clinical practice guidelines [10,11]. These system-level strategies have intended to shift patient and prescriber behavior toward prioritizing non-pharmacological and non-opioid pain management, prescribing opioids at the lowest effective dose for the shortest possible duration, and deprescribing opioids when the risks of continued use outweigh the potential benefits [11–14].

*Deprescribing* is the patient-centered process of dose reducing or discontinuing a medication that is no longer appropriate or necessary, with the intention of reducing iatrogenic harm [15]. Opioids have been identified as a key therapeutic target for deprescribing (also commonly referred to as ‘tapering’), owing to an unfavorable benefit-risk profile when used long-term for chronic non-cancer pain [12,16]. Evidence suggests that opioids do not offer long-term, clinically meaningful improvements in pain or function compared to placebo or non-opioid alternatives for chronic non-cancer pain [17,18], and cause adverse effects in 80% of people [19]. Despite this, approximately one-quarter of individuals with chronic non-cancer pain take opioid analgesics [20,21]. Patient-focused opioid deprescribing interventions, while heterogenous in their approach and outcomes, generally appear to be effective in reducing opioid consumption without worsening pain or physical function [22–24], and research indicates that most individuals would be willing to have a medication deprescribed if their doctor said it was possible [25]. The question arises then: why is opioid deprescribing not being routinely implemented in practice?

Opioids have been identified as a particularly difficult medication class to deprescribe, owing to medication, patient, prescriber and health system level barriers [16,26,27]. Factors such as physical and psychological opioid dependence, perceived ongoing benefit of opioid therapy, as well as a lack of effective, acceptable, and accessible alternatives for the management of persistent pain, often contribute to patient resistance, hindering deprescribing efforts [27]. Further, deprescribing long-term opioids is often reported to be difficult and time-consuming, particularly for primary care clinicians who are often responsible for opioid prescribing [27]. While some of these challenges are intrinsic to the medication, patient or prescriber [16], others could be addressed by leveraging policy instruments, incentivizing deprescribing, and scaling initiatives to enhance their reach and impact [28,29]. Numerous policy initiatives have attempted to reduce opioid use and related harm [10,30], however, it remains unclear whether these measures effectively support *deprescribing* - a process fundamentally rooted in shared decision-making between individual patients and healthcare professionals [15].

In this article, we discuss whether and how the concept of opioid deprescribing can be operationalized at a policy level. We review the goals, challenges and consequences of prescription opioid policies, explore how they intersect with system-level factors, and propose pathways for developing and implementing future opioid deprescribing policies.

## 2. Prescription opioid control policies

Health policy is commonly defined as “*courses of action (and inaction) that affect the set of institutions, organizations, services and funding arrangements of the health and health care system*” [31]. Health policy decisions are made in both the public government and private sectors; are influenced by, and influence other sectors beyond health; and are shaped by the sociocultural and political context in which policies function [31]. While this is a broad definition, in the context of opioids, policy often involves decisions that affect and govern access to, and safe use of opioids. The perception, acceptability and use of opioids is driven by sociocultural, political, medical, and health system contexts, which influence policies, and in turn, continue to shape attitudes toward opioids [32]. Such policies are influenced by the political goals and priorities of a jurisdiction, which play a significant role in determining the allocation of funding and resources (e.g., for pain treatments or harm reduction initiatives), and shape discourse around the use of opioids and other drugs [33,34].

The goals for policies concerning opioids are generally to improve access and safe use for individuals with therapeutic need, and to prevent or reduce non-medical use. To achieve this, policies have been typically implemented at the national or subnational level, often using regulatory and financial instruments, enabling consistency to be applied across relevant jurisdictions [11]. These policies are not necessarily always about *reducing* opioid use; however, in the past few decades, this has generally been the goal [35], given the phenomena of opioid overuse, poisonings, and related harms observed in the Global North. Such strategies have been referred to as ‘opioid control policies’ as they aim to control access through legal restrictions on distribution, or by influencing the decisions of health professionals as the gatekeepers to lawful opioid access [36,37].

## 3. Opioid control policies and opioid deprescribing: what’s the difference?

While opioid deprescribing and opioid control policies share a common goal – minimizing opioid-related harm – their approaches fundamentally differ. Deprescribing is typically a patient-level intervention that emphasizes individualized care, where the decision to reduce or cease opioids is guided by a careful assessment of the likely benefits and risks for a specific person, taking into consideration their clinical characteristics, goals, and preferences [15]. The motivation behind deprescribing is typically to alleviate medication burden and harm while maintaining or improving quality of life.

In contrast, opioid control policies, by their very nature, target populations rather than individuals; they are designed to reduce harm at a health system and organizational level by promoting opioid prescribing, dispensing and consumption behaviors that are deemed appropriate [10]. However, care that is deemed broadly appropriate at a population level may not always align with what is best for an individual. Accordingly, the implementation of opioid control policies has resulted in unintended consequences, such as reduced access to care and negative treatment experiences for some, including worsened pain, mental health crises, and even suicide [10,38]. Policies that mandate opioid reduction without accounting for patient agency, clinical needs, and preferences, directly contradict the fundamental principles of deprescribing.

So, is it possible for the defining features of deprescribing, such as patient-centeredness, individualization and shared decision making, to be effectively translated and embedded into policy? To answer this question, we examine several examples of policy initiatives (not exhaustive) that aim to support appropriate opioid use, describing their goals, promises, and pitfalls (with further characteristics summarized in Table 1).

**Table 1.** Classification of opioid policies by feature.

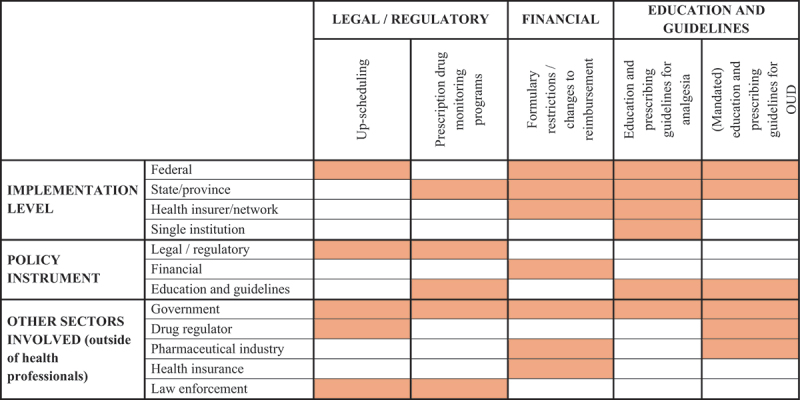

### 3.1. Up-scheduling

Scheduling has been employed as a legislative tool to regulate opioid access by reclassifying medications into more restrictive categories, a process known as “up-scheduling” [39]. This approach limits the availability of opioids compared to other substances, though access levels for the same drug can vary across jurisdictions. The primary objectives of up-scheduling are to reduce non-medical opioid use and diversion, encourage greater prescriber oversight of opioid use, and raise awareness among healthcare providers and the public about the potential risks associated with these medications [40]. However, restricting access to opioids in this manner can lead to substitutive prescribing and use of other analgesics, illicit opioids, or anxiolytic/sedative medications that may be a less favorable or appropriate option, equally or more harmful, or not to a patient’s preference [41,42]. Examples of therapeutic opioids that have been up-scheduled within the last two decades include hydrocodone and tramadol in the United States (US), and codeine in Australia. In the case of hydrocodone, a review of studies examining the impact of the scheduling change found reductions in indicators of hydrocodone prescribing and increases in the use of other opioids, particularly codeine and to a lesser extent tramadol (likely constrained by its own reclassification as a Schedule IV substance in 2014) [40]. However, mechanisms of any substitution were unclear and context dependent [40]. For codeine in Australia, following up-scheduling there was a decrease in codeine prescribing, sales, and calls to a jurisdictional poison center [40]. Additionally, studies found no increase, or small increases in other pharmaceutical opioid prescriptions [40].

### 3.2. Prescription drug monitoring programs (PDMPs)

PDMPs are electronic databases designed to track, often in real-time, the prescribing and supply of opioids and other controlled substances (e.g., benzodiazepines, stimulants) to reduce diversion and drug-seeking behavior, and to promote appropriate prescribing and dispensing [43]. Evidence suggests that PDMPs can decrease the number of adverse opioid-related events, increase communication among healthcare workers and patients and modify healthcare practitioners’ approach toward their opioid prescribed patients [44]. To maximize the effectiveness of PDMPs, complementary policies have been introduced, including mandates requiring providers to use PDMPs, enabling data sharing between jurisdictions, and issuing unsolicited reports to providers on patients’ prescription histories. Mandatory PDMP use increases their effectiveness [45]. However, similar to scheduling policies, PDMPs can lead to unintended consequences. For example, restrictions or changes to therapy may leave patients with limited analgesic options. Research has also linked the implementation of PDMP policies to increased heroin overdoses [46]. Additionally, PDMP use can sometimes strain the trust between patients and providers, leading to the undertreatment of pain and psychological and physical distress [47,48].

### 3.3. Funding instruments

Policymakers have used funding instruments, such as the inclusion of specific opioids for specific populations and indications on drug formularies. This sends a cost signal to patients and providers about what is considered appropriate use and for whom [49,50]. Additionally, it may also aid with reducing some inequities in accessing treatment [51,52]. In 2020, the Australian Pharmaceutical Benefit Scheme (PBS) introduced changes to the way in which government-subsidized prescription opioids were supplied for patients with severe pain; restricting the number of repeat prescriptions, reducing pack sizes, and increasing the level of authorization required to prescribe some opioids [50]. Tightened PBS restrictions led to a decrease in PBS subsidized opioid use [53], however, restrictions were met with mixed responses from the community. For some, it led to an extra burden of needing to return to their prescribers to get additional prescriptions or being driven to the pay privately out-of-pocket to obtain pharmacological pain relief [54]. One in three chronic pain sufferers reported difficulty accessing ongoing opioid prescriptions following these changes [55].

### 3.4. Education and guidelines

Education and guidelines have been established for patients and providers on what is considered appropriate and safe use of opioids, providing guiding principles and recommendations for practice [11–14]. In the US, the 2023 Medication Access and Training Expansion (MATE) Act required DEA-registered healthcare professionals who prescribe controlled substances to complete an educational program on the treatment and management of patients with opioid or other substance use disorders. Additionally, the US National Centres for Disease Control (CDC) Guideline for Prescribing Opioids for Chronic Pain, first released in 2016, endeavored to promote more judicious opioid use [56]. However, some policies and practices purportedly derived from the guideline deviated from, or misapplied guideline recommendations [57], encouraging inflexible application of dosage and duration thresholds (interpreting them as hard limits), and inciting abrupt tapering or cessation [58]. This led to unintended consequences such as termination of care and increased incidence of suicide and illicit opioid use [59–61].

## 4. Factors affecting the success of opioid control policies

Following the implementation of opioid control policies, there has been a decline in opioid use across many countries [35]. However, this reduction has not consistently translated into lower opioid-related mortality, or meaningful improvements in adherence to guideline-concordant care [30,62]. This disconnect is likely influenced by the increased availability of potent synthetic opioids like fentanyl [63], as well as inadequate access to effective pain management, which may drive some patients to use illicit substances [37]. While the potential risks of these opioid control policies are now evident, it remains challenging to predict which future policies will strike the right balance between ensuring safe opioid use and minimizing unintended harm.

**Figure 1.**
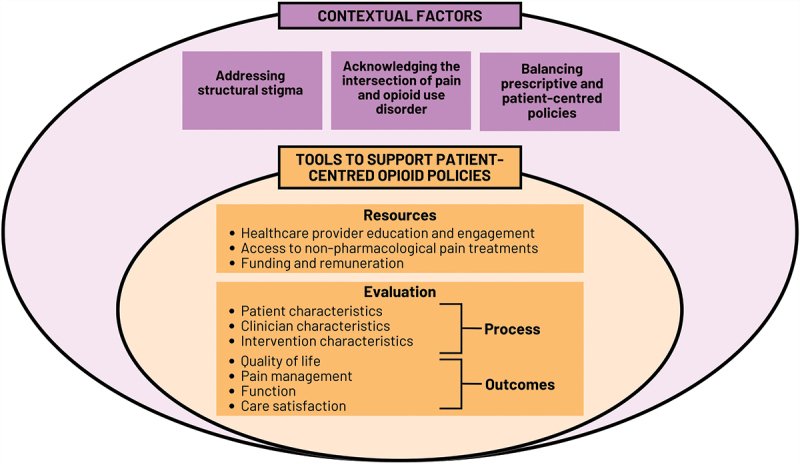
Conceptual summary of contextual factors influencing opioid control policy outcomes and tools to support patient-centred opioid deprescribing policies.

We propose that several key system-level factors inherently influence the success and consequences of opioid control policies. In the following section, we outline major challenges associated with developing and implementing opioid policies and identify critical action points that require addressing to facilitate more patient-centered opioid deprescribing policies (summarized in Figure 1).

### 4.1. Structural stigma and the delivery of opioid-based treatments

One of the major challenges for people taking opioids for pain and/or OUD, and for people who use drugs, is the stigma and marginalization they encounter in society and within the healthcare system. This in turn leads to barriers to accessing and receiving care, where individuals may experience delays in accurate diagnoses and effective treatment (for both conditions requiring opioid therapy and those not); inadequate, discriminatory, and/or harmful care, and thus, decreased therapeutic effectiveness and retention [64,65]. Opioid-related stigma is a complex phenomenon; previous work has demonstrated the different ways in which opioid-related stigma is constructed and experienced: interpersonally and structurally toward people using opioids for managing OUD and chronic pain; within the healthcare system; and self-stigma [66]. This is highly relevant to opioid policy; as policy exists within a sociocultural and political context. Therefore, structural stigma, particularly in the healthcare system, needs to be examined for how it poses a challenge to the current and future development and implementation of opioid deprescribing policy.

Structural stigma – characterized by societal conditions, cultural norms, and institutional policies that limit opportunities and well-being for stigmatized groups – profoundly shapes the experiences of individuals who use opioids [67]. Healthcare providers’ negative attitudes toward individuals with substance use disorders are well-documented, with these patients often perceived less favorably compared to those with other mental health conditions [68,69]. Recent findings extend this bias to patients who take prescription opioids, with primary care physicians demonstrating negative attitudes that perpetuate stigma and hinder trust in provider-patient relationships [70]. For many of these patients, the label of “addict” has become a dominant social identity, overshadowing their experiences and needs, and undermining their sense of agency in decisions about pain management and opioid use [71,72]. More specific to opioid deprescribing, the development of opioid tapering initiatives has been rooted in societal-level events demonstrating the harms of opioids, for example, opioid overdose crises stemming from overprescribing in countries like the US and Canada. This laid the foundation for the acceptability, development, and implementation of measures to reduce and control the supply and accessibility of opioids. While public discourse emphasizes reducing stigma and promoting patient-centered care, the implementation of opioid control policies often contradicts these ideals. Research in the US with a population of chronic non-cancer pain patients, demonstrated their negative experiences of opioid tapering initiatives [73]. Patients viewed opioid tapers to be a result of broader health system, political, and sociocultural crackdown on the use of opioids for chronic pain, did not experience these initiatives to be patient-centered, and felt ignored and stigmatized in the context of policies purportedly addressing the opioid crisis [73].

Opioids are amongst the most highly regulated medicines for therapeutic use, with structural mechanisms in place to maintain this. Previous work has identified a “regulatory cascade” phenomenon regarding opioids, where stakeholders at all levels throughout the healthcare system contribute to, or are subject to the decreased accessibility of opioids and ‘control’ from actors further upstream in the system [32]. While this has been applied more specifically to opioid agonist therapy to manage OUD, we can see similarities with the ways in which this approach influences how opioids are prescribed for analgesia. Healthcare professionals may encounter additional oversight of their practice – whether from regulators, auditors or their peers – in the form of prescribing/dispensing reviews, or mandated review of prescription drug monitoring data. They may be subject to additional education, training or bureaucratic requirements that foster an environment of paternalism and fear of wrongdoing. These requirements also contribute to fewer healthcare providers choosing to deliver care for people using opioids [32], thus continuing the siloing of chronic pain and OUD care, and perpetuation of stigma in the healthcare system. This burden may then be passed onto patients, where they are subject to suspicion of drug-seeking behavior and measures to counteract this, such as urine drug testing, entering treatment contracts, or having risk stratification tools applied. Requiring opioid contracts or routine urine drug testing creates a dynamic that would be unthinkable in managing other chronic conditions, where patients are not asked to sign agreements to ensure treatment adherence. Although opioids have unique risks that may necessitate tailored approaches, patients with opioid-related conditions are no less deserving of empathy, dignity, and care than those managing other chronic illnesses. These measures have been perceived by people with chronic pain and/or OUD who use opioids as punitive and dehumanizing, further eroding trust in the healthcare system [71].

### 4.2. Acknowledging the intersection of pain and OUD

The intersection of pain and OUD presents a significant challenge in the clinical management of people who take opioids, as well as opioid-related research and policy. Individuals receiving opioid agonist therapy for OUD report a high prevalence of pain, often at greater rates than the general population [74]. Similarly, a notable proportion of people who take prescription opioids develop dependency [75–77]. However, current clinical practice guidelines largely conceptualize pain and OUD as separate entities, neglecting the continuum and reality that many navigate. For example, for chronic non-cancer pain, guidelines suggest deprescribing long-term opioids when the harms of continuation outweigh the benefits [12,13]. Conversely, for individuals with severe OUD, guidelines caution against deprescribing, emphasizing the necessity of ongoing opioid agonist therapy [12,78]. While both respective recommendations are evidence-informed, their application may be complicated by the realities of real-world clinical practice, where patients rarely fit neatly into discrete categories of ‘pain’ or ‘OUD,’ making it challenging for clinicians and patients to navigate care.

The self-perception of people who take opioids adds another layer of complexity. Some may see themselves primarily as individuals managing pain, while others may identify with, or resist, the label of opioid dependence or a substance use disorder, influencing how they approach and engage with deprescribing conversations [26]. Current policy approaches focused primarily on opioid reduction, risk oversimplifying the diverse realities of these patients, limiting the ability of healthcare providers and systems to address their unique needs.

The dichotomy between opioid management for pain and OUD is also mirrored in regulation, where opioids prescribed for pain may be subject to fewer restrictions than those used for opioid agonist therapy [79,80]. In many jurisdictions, regulations govern who can prescribe and dispense opioids for opioid agonist therapy, including regulated and/or mandated education, training, guidelines, or standards to follow and maintain. Consequently, these restrictions can limit treatment access for individuals with OUD. In response, deregulation measures have been introduced in some countries, such as the removal of the historical ‘X waiver’ requirement for prescribing buprenorphine in the United States [81], and the federal exemption requirement for prescribing methadone in Canada [82]. These licensing requirements were often accompanied by additional stipulations, including preceptorship, registration with regional regulatory colleges, and audits or practice reviews. Following the regulatory change in Canada, a number of provinces demonstrated growth in the number of OAT prescribers [79].

### 4.3. Balancing prescriptive and patient-centred policies

Part of the reason why system-level efforts to curb opioid prescribing produce unintended consequences, is that they fail to account for the heterogeneity and diverse needs of individuals who take opioids. Not only are opioids prescribed for multiple indications, but people who consume opioids are often a complex patient cohort, with high prevalence of comorbidities, including mental health disorders [83]. Some of these people are the most vulnerable and perhaps the least adaptable to changes in their care delivery, further underscoring the need for shared decision-making when developing individualized tapering plans. However, this patient-centered approach becomes difficult to achieve under policies that mandate specific actions, or impose rigid, one-size-fits-all approaches. Prescribers have expressed dissatisfaction with overly paternalistic and unnecessarily restrictive opioid policy initiatives. In a qualitative study of Australian primary care physicians, participants reported feeling victimized by reductive opioid policy approaches, which fostered a climate of fear surrounding potential litigation for inappropriate prescribing [84]. The ramification is that some prescribers may avoid initiating or continuing opioids altogether. Such defensive practices risk eroding the therapeutic relationships essential for shared decision-making and open communication – both essential for navigating the complexities of opioid deprescribing. Further, policies that prioritize rigid mandates over clinical discretion threaten to exacerbate existing vulnerabilities, undermine the appropriate management of comorbidities (e.g., mental health disorders), and compound structural stigma, further marginalizing those most in need of tailored, compassionate care.

Quality-promoting initiatives, such as guidelines, may be more acceptable to end-users compared to punitive measures or mandates which restrict individual choice [84]. The Australian evidence-based clinical practice guideline for deprescribing opioid analgesics underscores the importance of patient engagement and shared decision-making throughout the deprescribing process [12]. It also acknowledges that opioid dose reduction or cessation may not be suitable for all patients, emphasizing the need for individualized care [12]. However, the implementation of guideline recommendations remains non-obligatory, meaning that their impact may be less immediate or pronounced [85,86]. Moreover, less directive recommendations, designed to accommodate diverse individual circumstances, may be perceived as less actionable [87].

The tension between flexibility and specificity in guidelines is exemplified by the evolution of the CDC opioid prescribing guidelines in the US. The 2016 guideline included dose thresholds based on epidemiological evidence linking higher opioid doses to increased risks of overdose and death [56]. As these thresholds were operationalized as rigid dosing limits by some clinicians, policymakers, and payers, in the 2022 update, dose and duration thresholds were removed, shifting to an individualized, shared decision-making approach [13]. While this shift was welcomed by advocates of patient-centered care, critics argue that the lack of specificity may lead to inaction, potentially leaving some patients on long-term opioid therapy at risk of harm [88]. Given the low-certainty evidence base for opioid deprescribing at present [22–24], clinical practice guidelines play a vital role in translating complex evidence into actionable recommendations. Striking the right balance between flexibility and clear directives is essential to ensure that guidelines, and policies more broadly, are both practical and effective, guiding clinicians to deliver care that is patient-centered but enables mitigation of opioid-related risks.

## 5. System-level changes to facilitate the full potential of opioid deprescribing

A large cluster randomized clinical trial conducted in the Canadian province of Manitoba was published last year; the Trial Applying Policy to Eliminate or Reduce Inappropriate Narcotics in the General Population (TAPERING) [89]. This trial investigated whether a government-led, mailed, direct-to-consumer educational brochure could reduce prescription opioid use among community-dwelling adults with long-term opioid prescriptions [89]. The trial design drew inspiration from a previously successful direct-to-patient education intervention, which empowered individuals to deprescribe sedative medications [90,91]. By partnering with the government, the TAPERING trial targeted all eligible people within the jurisdiction, however, it found no significant difference in the number of people filling opioid prescriptions within six months between those who received the brochure and those who did not [89]. The authors offer several reasons for the intervention’s lack of impact on the primary end point. Below, we explore these reasons and present recommendations for system-level supports that may address or ameliorate identified barriers.

### 5.1. Healthcare provider education and engagement

The educational brochure in TAPERING advised patients to talk to their healthcare professional about opioid use and pain management. However, it was noted that many health professionals at the time had limited self-efficacy to deprescribe opioids [92]. To ensure that health professionals are equipped to engage in deprescribing conversations and support patients to reduce their therapies if appropriate, healthcare providers need to be adequately trained in opioid deprescribing, pain management, and shared decision-making. System-level support could involve integrating these topics into clinical school curricula, offering continuing professional development programs, and providing specialized training, (such as the MATE Act described earlier). This would enhance clinician confidence and skills in engaging patients and reduce stigma in the healthcare system, both essential for effective opioid deprescribing.

### 5.2. Access to non-pharmacological treatments

The trial authors postulated that individuals living in nonurban areas may have had difficulty accessing the multidisciplinary pain clinics or non-pharmacologic treatments referenced in the brochure [89]. Multidisciplinary care appears to be the most effective intervention for opioid deprescribing [22], but the reality is that many patients cannot access these services. Long wait times, regional inaccessibility, and high out-of-pocket costs are significant barriers to access [16,26,93]. To address this, governments must invest in infrastructure that supports comprehensive pain management, expanding access to multidisciplinary services or multimodal medical, pharmacy, psychological, and physiotherapy expertise. The educational brochure in TAPERING was a low-cost and light-touch intervention, whereas other recent clinical trials of opioid reduction interventions employed at the patient-level that have been more complex and time-intensive, have demonstrated success in both medication reduction and clinical outcomes [94,95]. However, scaling these types of interventions across entire health systems is hindered by resource limitations and insufficient infrastructure.

### 5.3. Funding and remuneration

The intervention brochure in the TAPERING trial was mailed directly to patients, rather than being delivered by a healthcare professional with whom the patient had an established relationship [89]. In contrast, pharmacists were involved in education delivery and played a crucial role in previous successful deprescribing trials for sedatives [91]. To incentivize healthcare professionals to engage in opioid deprescribing, formalized and sufficient funding and remuneration is required. In fee-for-service payment systems, clinicians may not be adequately compensated for the time-intensive activities involved in deprescribing, creating a financial disincentive; alternatively, they may be better compensated for other clinical activities at the expense of deprescribing processes and activities. However, introducing incentives for deprescribing carries risks: if poorly designed, they may encourage abrupt or unsolicited opioid tapering, potentially causing patient harm. Implementing remuneration models that reward healthcare professionals for efforts like extended consultations, collaborative care, and follow-ups may help bridge this gap without encouraging unsafe opioid reduction practices. Additionally, investing in educating, training and remunerating non-physician clinicians, such as pharmacists and nurses, to actively participate in deprescribing could alleviate the burden on primary care physicians and promote a team-based approach to care.

## 6. Changing how we evaluate opioid and/or deprescribing policies

High-certainty evidence on the outcomes of opioid deprescribing is lacking [22]. Similarly, there are deficiencies in study design rigor examining opioid control policies [11]. If we are to advocate for evidence-informed practice and policy reforms (which we do), evidence on what works, for whom, and why is needed.

To date, evaluations of opioid control policies have often neglected measures of process, patient-centered outcomes, or the experiences of prescribers [38]. Rather, they have typically focused on monitoring prescription patterns (i.e., opioid reduction rates) and safety metrics, such as mortality. While informative, this approach overlooks critical determinants and outcomes, such as patient access to opioids, clinical outcomes, and the processes through which healthcare organizations implement deprescribing practices. A shift is needed toward more holistic and patient-centered evaluation, examining outcomes such as quality of life, function, pain management, and satisfaction with care. Arguably, these outcomes are more difficult to measure, but they are vital for understanding the true impact of policies. This aligns with the recommendations for outcome measurement for deprescribing intervention studies [96], as well as the core outcome measures for chronic pain clinical trials, outlined in the Initiative on Methods, Measurement, and Pain Assessment in Clinical Trials (IMMPACT) statement [97]. Linking opioid prescribing data with other healthcare data is also critical for examining intended and unintended consequences of opioid-control policies. Collaborations among governments, data providers, researchers, and public health officials are necessary to ensure that policies can be coupled with thorough evaluations.

Similarly, understanding the *process* of opioid reductions is essential for developing meaningful and evidence-based policy interventions. This involves examining:

- **patient characteristics**, such as clinical characteristics, social capital and support networks, knowledge, self-efficacy and health beliefs,
- **healthcare professional characteristics**, such as knowledge and competence related to opioid management and pain care; confidence and ability to navigate professional pressures,
- **intervention characteristics** (i.e., specific steps taken to alter care), such as the flexibility of the policy; whether policies are standardized or allow for individualization; the provision of other co-interventions to change pain care and analgesic prescribing; and whether proposed alternatives to opioids are appropriate for the patient, healthcare professional, and setting [38,98].

Ultimately, developing evidence-informed solutions requires engaging and retaining patients in opioid research to account for the lived experiences and needs of those most impacted. There has been a recent emphasis on co-designing policies with input from all key stakeholders. However, this may be methodologically and ethically challenging given the vulnerability and marginalization of these populations [38]. Additionally, unique aspects of de-implementing inappropriate opioid use differentiate it from implementing evidence-based interventions [98]. Policymakers and implementation scientists can address these deficiencies by applying an implementation science framework, which offers a systematic approach to studying and improving the processes of de-implementation.

Policy analyses of other jurisdictions’ opioid deprescribing policies and strategies should also be conducted to critically examine how contextual epidemiological, health system, political, and sociocultural factors influence these policies’ existence, development, and implementation. Successes in one jurisdiction may not translate neatly to another, and care should be taken before outright adopting a policy without consideration of contextually relevant needs. This approach may be particularly pertinent when we take a global perspective, as in many countries in the world, there is an impetus to increase access to opioids. In contexts where many suffer due to the unavailability of opioids, deprescribing is likely not the priority [99]. However, as access expands, it would be prudent for policy actors to learn from other jurisdictions’ policy successes and failures, how they occurred, and put measures and safeguards in place to prevent downstream overprescription and subsequent harm.

## 7. Conclusion

In conclusion, this article has reviewed the goals, challenges, and consequences of opioid control policies and explored their intersection with system-level factors. In doing so, it has outlined proposed pathways to guide the development and implementation of future patient-centered opioid deprescribing policies.

## 8. Future perspective

Existing opioid control policy interventions have not addressed all determinants of inappropriate opioid prescribing and usage [62]. We assert that opioid deprescribing policies have the potential to be patient-centered. Achieving this vision in practice is undeniably complex, requiring careful consideration of how policies are selected, communicated, developed, and implemented.

While regulatory tools like restrictions can be effective, they also risk being overly blunt, limiting access to care, and reinforcing opioid-related stigma. On the other hand, non-mandatory strategies such as guidelines or education alone may lack the necessary impact. The key to effective deprescribing lies in striking a balance between providing incentives and maintaining flexibility – ensuring decisions are tailored to each patient’s individual goals, values, and needs, while also being feasible to implement in real-world healthcare settings. Central to this balance is embedding patient autonomy and informed consent at every stage of the deprescribing process, alongside ensuring clinician acceptability. Achieving a truly patient-centered approach requires prioritizing patient and clinician involvement in policymaking, design, and implementation, as well as throughout the clinical process.

Opioid deprescribing is but one element of a broader, multifaceted challenge in managing individuals with pain. Given the complexity of opioid deprescribing at both the patient and system level, robust clinical and system supports are essential. Ongoing evaluation and adaptation of trialed strategies will be key to determining and ensuring their success. Additionally, advancing pain prevention and management strategies will help reduce the burden on individuals, ensuring that opioid deprescribing efforts are part of a comprehensive, sustainable approach to chronic pain care.

## Article highlights

- Opioids are used for a range of clinical indications, including pain and opioid use disorder; however, the risk of opioid-related harm has led to their classification as high-risk medications that require policies to mitigate negative consequences for individuals and populations.
- Deprescribing is the patient-centered process of dose reducing or discontinuing a medication that is no longer appropriate or necessary, with the intention of reducing harm.
- Opioids are a key target for deprescribing, and while individual- and system-level barriers to deprescribing have been reported, there have also been calls to operationalize opioid deprescribing through implementing deprescribing policies.
- There are natural tensions between deprescribing as a tailored patient-centered process of shared decision-making between individual patients and healthcare professionals, and opioid policies as broader system-level strategies that are designed to reduce harm at a health system and organizational level.
- The success and consequences of opioid policies are shaped by sociocultural, political, medical, and health system contexts; in the case of developing and implementing more patient-centered opioid deprescribing policies, we propose system-level factors and strategies to facilitate this: addressing structural stigma, acknowledging the intersection of pain and opioid use disorder, and balancing prescriptive and patient-centered policies.
- Subsequently, these factors should guide the deployment of resources and policy evaluation (e.g., clinician training and remuneration, support for complex patients, consideration of outcomes important to patients) to support patient-centered opioid deprescribing policies.
- Opioid deprescribing policies have the potential to be an important tool in reducing opioid-related harms from a patient-centered lens; however, given the inherent complexity of opioid use at the patient- and system-level, this will require careful consideration of policy design, development, and implementation.
